# Field-based evidence for consistent responses of bacterial communities to copper contamination in two contrasting agricultural soils

**DOI:** 10.3389/fmicb.2015.00031

**Published:** 2015-02-02

**Authors:** Jing Li, Yi-Bing Ma, Hang-Wei Hu, Jun-Tao Wang, Yu-Rong Liu, Ji-Zheng He

**Affiliations:** ^1^State Key Laboratory of Urban and Regional Ecology, Research Center for Eco-Environmental Sciences, Chinese Academy of SciencesBeijing, China; ^2^College of Resources and Environment, University of Chinese Academy of SciencesBeijing, China; ^3^National Soil Fertility and Fertilizer Effects Long-term Monitoring Network, Institute of Agricultural Resources and Regional Planning, Chinese Academy of Agricultural SciencesBeijing, China; ^4^Faculty of Veterinary and Agricultural Sciences, The University of MelbourneParkville, VIC, Australia

**Keywords:** copper contamination, soil bacterial community, diversity, abundance, community composition, soil microbial biomass carbon, field experiment

## Abstract

Copper contamination on China's arable land could pose severe economic, ecological and healthy consequences in the coming decades. As the drivers in maintaining ecosystem functioning, the responses of soil microorganisms to long-term copper contamination in different soil ecosystems are still debated. This study investigated the impacts of copper gradients on soil bacterial communities in two agricultural fields with contrasting soil properties. Our results revealed consistent reduction in soil microbial biomass carbon (SMBC) with increasing copper levels in both soils, coupled by significant declines in bacterial abundance in most cases. Despite of contrasting bacterial community structures between the two soils, the bacterial diversity in the copper-contaminated soils showed considerably decreasing patterns when copper levels elevated. High-throughput sequencing revealed copper selection for major bacterial guilds, in particular, *Actinobacteria* showed tolerance, while *Acidobacteria* and *Chloroflexi* were highly sensitive to copper. The thresholds that bacterial communities changed sharply were 800 and 200 added copper mg kg^−1^ in the fluvo-aquic soil and red soil, respectively, which were similar to the toxicity thresholds (EC50 values) characterized by SMBC. Structural equation model (SEM) analysis ascertained that the shifts of bacterial community composition and diversity were closely related with the changes of SMBC in both soils. Our results provide field-based evidence that copper contamination exhibits consistently negative impacts on soil bacterial communities, and the shifts of bacterial communities could have largely determined the variations of the microbial biomass.

## Introduction

Soils represent the largest sink for copper released into the environment by anthropogenic activities, such as sewage irrigation, mining activities, municipal waste disposal, and intensive use of pesticides and herbicides (Smith, 2009; Zhuang et al., 2009). Approximately 3.4 million ton copper is released to the terrestrial surface every year (Zhou et al., 2000). High copper concentrations have been found in paddy soils (109–1313 mg kg^−1^) near mining sites (Zhuang et al., 2009), and vineyard soils increasing from background levels of 10 mg kg^−1^ to approximately 250 mg kg^−1^ with intensive use of copper-based fungicides (Pietrzak and McPhail, 2004). Moreover, it was predicted that up to 20% of China's arable lands were contaminated by heavy metals and the areas will continuously expand due to the intensification of human activities in the coming decades, especially the agricultural soils (Li et al., 2014b).

Microorganisms are highly diverse and ubiquitous in soil ecosystems, and participate in a variety of key ecosystem functions such as nutrient cycling, feedback responses to climate change and biomass production. A stable microbial community contributes essentially to stabilizing soil structure and maintaining soil ecosystem services (Bissett et al., 2013). Moderate level of copper regarded as a micronutrient element is necessary for microorganisms to carry out the normal metabolic activities (Giller et al., 2009). However, excessive inputs of it into soil ecosystems could persist for a long time after their introduction and cause negative and toxic effects on the inhabitant microorganisms, and thereby affect the critical functioning they mediate (Giller et al., 2009). Therefore, understanding responses of soil microbial assemblages to copper contamination is essential to counteract its negative effect on ecosystem functions and services.

Despite a generally accepted view that the accumulation of copper might change soil microbial communities and affect microbial activities, most of the experiments were performed in short-term laboratory incubations with single soil type (Girvan et al., 2005; Lejon et al., 2008). However, the bioavailability and toxicity of copper might change over time due to aging and equilibration effects (Ma et al., 2006; Singh et al., 2014), so the long-term impacts of copper contamination on soil microorganisms differed across different sites. It is necessary to study whether the responses of soil microbes to long-term copper contamination are consistent under contrasting field conditions.

The goal of this study was to elucidate how the soil bacterial abundance, diversity, and community composition respond to different intensities of copper contamination under field conditions, and try to find the relationship between these changes and microbial community function. Soils were collected from two field experimental sites in China which have shown negative impacts of copper contamination on maize grain yield (Guo et al., 2010). We hypothesized that: (1) the bacterial communities could be significantly affected by long-term copper contamination in both contrasting field sites; (2) these strong effects might select copper-sensitive and–tolerant bacterial groups; (3) the shifts of bacterial communities might be observed along the copper gradients and these changes might influence the microbial functioning, such as the soil microbial biomass carbon (SMBC).

## Materials and methods

### Field description and soil sampling

Soil samples were collected from two long-term experimental stations located in Dezhou (37.33° N, 116.63° E), Shandong province, and Qiyang (26.75° N, 111.88° E), Hunan province, China. Dezhou is characterized by a temperate continental climate with a mean annual temperature of 12.9°C and a mean annual rainfall of 547.5 mm. The soil in Dezhou is classified as fluvo-aquic soil according to the Chinese soil taxonomy with a ratio of clay: silt: sand (18:18:64) and a pH value of 7.9. In the Dezhou site, copper chloride powders were mixed thoroughly with surface soil and then applied back into the plots in July 2007. There were eight copper treatments, with four replicated plots for each treatment, and the copper levels were 0, 50, 100, 200, 400, 800, 1600, and 3200 mg kg^−1^ soil. The Qiyang site is characterized by a subtropical monsoon climate with a mean annual temperature of 18.1°C and a mean annual rainfall of 1407.5 mm. The soil in Qiyang is classified as red soil with a ratio of clay: silt: sand (46:35:19) and a pH value of 4.3. The copper chloride powders were added into Qiyang soils in July 2007. The copper levels were 0, 12.5, 25, 50, 100, 200, 400, and 800 mg kg^−1^ soil, with four replicated plots for each treatment.

The both sites had been planted with maize-wheat rotations under conventional agricultural management practices since 2007. Totally 128 soil samples were collected from the top 10 cm by mixing five soil cores for each plot in July 2011 and July 2012, respectively. Soil samples were stored at 4°C before analyses of physicochemical properties and −80°C prior to DNA extraction.

### Soil properties analyses

Soil pH was measured with a soil to water ratio of 1:2.5 using a pH meter. Soil moisture content was determined after oven drying at 105°C for 8 h. Soil organic matter (SOM) was measured by the K_2_Cr_2_O_7_ oxidation-reduction colorimetric method. Total nitrogen (TN) was measured by a CN analyzer (Vario EL III, Elementar, Germany). The copper extracted with 0.11 M CH_3_COOH was determined on ICP-OES (PerkinElmer, Waltham, MA, USA). The detailed characteristics of the soils are listed in Table 1.

**Table 1:** **Soil characteristics of the examined samples collected from Dezhou and Qiyang**.

**Soil type**	**Treatment**	**pH**	**H_2_O (%)**	**SOM (g kg^−1^)**	**TN (g kg^−1^)**	**Cu added (mg kg^−1^)**	**Extractable Cu (mg kg^−1^)**
Fluvo-aquic soil (Dezhou)	Cu0	7.8	14.1	13.4	0.46	0	0.5
	Cu50	7.7	14.2	11.3	0.50	50	3.6
	Cu100	8.1	13.6	12.3	0.46	100	12.9
	Cu200	8.0	15.8	11.5	0.44	200	38.7
	Cu400	8.0	15.8	12.8	0.40	400	134.6
	Cu800	7.9	15.5	12.3	0.44	800	406.0
	Cu1600	7.8	16.7	11.8	0.40	1600	951.8
	Cu3200	7.7	16.8	12.0	0.38	3200	1205.3
Red soil (Qiyang)	Cu0	4.3	20.2	16.2	0.92	0	1.4
	Cu12.5	4.3	20.5	15.6	0.92	12.5	5.3
	Cu25	4.3	20.0	15.9	0.86	25	10.5
	Cu50	4.1	19.3	15.6	0.88	50	22.0
	Cu100	4.1	18.6	13.7	0.82	100	48.6
	Cu200	4.2	10.1	15.0	0.86	200	96.7
	Cu400	4.3	11.3	15.3	0.86	400	211.3
	Cu800	4.4	12.7	16.5	0.78	800	377.9

### Determination of soil microbial biomass carbon (SMBC)

Soils were pre-incubated in semi-sealed vessels at 25°C for 15 days with 40% water-holding capacities (WHC). After the pre-incubation, three replicates of the moist soil (equivalent to10 g oven-dry soil) were fumigated with ethanol-free CHCl_3_ for 24 h at 25°C in sealed desiccators containing water and soda-lime. The dissolved carbon of fumigated soils (E) was extracted by shaking for 30 min with 0.5 M K_2_SO_4_, and the dissolved carbon was also extracted from the other three replicates of non-fumigated soils (E_0_). The concentrations were determined on a Liqui TOC analyzer (Liqui, Elementar, Germany). SMBC = (*E* − *E*_0_)/k (*k* = 0.45) (Wu et al., 1990).

### DNA extraction and quantitative polymerase chain reaction (qPCR)

Soil DNA was extracted using MoBio Powersoil DNA Isolation Kit (MoBio Laboratories, Carlsbad, CA, USA) according to the manufacturer's protocol. The quantity and quality of the extracted DNA were examined using a NanoDrop^®^ ND-2000c UV-Vis spectrophotometer (NanoDrop Technologies, Wilmington, DE, USA).

Abundance of the bacterial 16S rRNA gene was determined by qPCR on an iCycler iQ 5 thermocycler (BioRad Laboratories, Hercules, CA, USA) using the primer pairs BACT1369F and PROK1492R with the probe TM1389F (Suzuki et al., 2000). Each reaction was performed in a 25 μl volume containing 12.5 μl Premix Ex Taq (Takara Biotechnology, Dalian, China), 0.25 μl of each primer (10 μM), 0.5 μl of probe (10 μM) and 1 μl of five-fold diluted DNA template (1–10 ng). Amplification conditions were as follows: 95°C for 10 s, 35 cycles of 15 s at 95°C and 1 min at 56°C. Standard curves were developed using ten-fold serial dilutions of plasmid containing correct insert of the bacterial 16S rRNA gene.

### PCR amplification and sequence processing

The V4 region of the bacterial 16S rRNA gene was amplified with the primers 515f and barcoded-806r which target a broad diversity of bacteria with few biases against particular groups (Bates et al., 2011). The PCR reactions in a 50 μl mixture contained 20 μl Premix Ex Taq (Takara Biotechnology), 0.4 μl of each primer (10 μM), 4 μl of five-fold diluted template DNA (1–10 ng) and 25.2 μl sterilized water. Thermal-cycling conditions were as follows: an initial denaturation of 3 min at 94°C, six touchdown cycles of 45 s at 94°C, 60 s from 65°C to 58°C, 70 s at 72°C, followed by 22 cycles of 45 s at 94°C, 60 s at 58°C, 60 s at 72°C with a final elongation of 72°C for 10 min. The PCR products were purified using a Wizard SV Gel and PCR Clean-up system (Promega, San Luis Obispo, CA, USA). The concentrations of the PCR products were fluorometrically quantified by the Qubit dsDNA HS Assay Kit (Invitrogen, Carlsbad, CA, USA) before being sequenced on the Miseq platform (Illumina, San Diego, CA, USA), at Novogene, Beijing, China.

Raw sequences were processed in QIIME 1.7.0 (Caporaso et al., 2010a). Sequences were quality trimmed and clustered into operational taxonomic units (OTUs) at a 97% identity threshold using uclust (Edgar, 2010). Representative sequences from individual OTUs were then aligned against the Greengenes core set (DeSantis et al., 2006) using PyNAST (Caporaso et al., 2010b). Taxonomic assignment was carried out with the RDP Classifier (Wang et al., 2007). Resampling for each sample according to the minimum sequence numbers was performed before the downstream analyses.

The principal coordinate analysis (PCoA) was used to visualize the Bray-Curtis dissimilarity matrices based on the 97% OTU level across different copper concentrations (Caporaso et al., 2010a). Diversity was characterized by calculating richness (OTU numbers, Shannon index) and evenness (Gini coefficient). The Gini coefficient (ranging from 0 to 1) is a value to assess the specific degree of evenness, and a higher Gini coefficient indicates lower evenness of a community (Wittebolle et al., 2009). The Gini coefficient was calculated by performing *ineq* package in R3.0.2 software (http://www.r-project.org/).

### Statistical analysis

The 16S rRNA gene copies were log-transformed prior to statistical analysis to satisfy the normality assumptions. One-Way analysis of variance (ANOVA) followed by Student-Newman-Keuls (homogeneous variance) and Welch's *t*-test (non-homogeneous variance) was conducted to compare SMBC, the bacterial abundance across different copper concentrations, and the relative abundances of different phyla between the two soils in SPSS 19.0 (IBM Co., Armonk, NY, USA). The EC50 values were calculated by logistic model using SigmaPlot 12.0 (Li et al., 2010). Distance-based multivariate linear model (DistLM) was utilized to analyze the relationships between the soil properties and the bacterial diversity as well as community compositions. Permutation multivariate analysis of variance (PerMANOVA) was used to evaluate the significance of the Bray-Curtis dissimilarity matrices across copper gradients. Boxplots plotted in R.3.0.2 were used to display the variations of the SMBC and abundance along copper gradients. Heat maps were generated to show differences in the bacterial community compositions based on the dominant phylum (with a relative abundance >5%) along the copper gradients. The heat map using the *gplots* package and PerMANOVA using the *vegan* package were performed in R.3.0.2. Spearman's rank test was used to assess the correlations between the abundance, Gini coefficient, OTU numbers, Shannon index, relative abundances of some groups, SMBC and copper concentrations. *P* < 0.05 was considered to be statistically significant.

Structural equation model (SEM) was constructed to investigate the direct and indirect effects of bacterial abundance, diversity, composition, and copper concentrations on microbial biomass. Diversity was characterized by Shannon index, and composition was characterized by first axis of PCoA using Bray–Curtis dissimilarity matrix. Based on a priori and theoretical knowledge, we assumed a conceptual model that the changes of bacterial abundance, diversity, composition and copper concentrations affect the variations of microbial biomass. Maximum likelihood estimation method was used to compare the SEM with the observation. Model adequacy was determined by χ^2^-tests, goodness-of-fit index (GFI), Akaike Information Criteria (AIC), root square mean errors of approximation (RMSEA), and we revised our conceptual model according to these indexes. Adequate model fits were indicated by a non-significant χ^2^-test (*P* > 0.05), high GFI (>0.90), low AIC, and low RMSEA (<0.05) (Grace, 2006). SEM analysis was performed using AMOS 17.0 (Amos Development Corporation, Meadville, PA, USA).

## Results

### Variations in soil characteristics and SMBC across different treatments

Soil pH varied from 7.7~8.1 and SOM ranged from 11.3~13.4 g kg^−1^ in the fluvo-aquic soil to 4.1~4.4 and 13.7~16.5 g kg^−1^, respectively, in the red soil (Table 1). Copper amendments consistently increased the extractable copper concentrations from 0.5 to 1205.3 mg kg^−1^ in the fluvo-aquic soil and 1.4 to 377.9 mg kg^−1^ in the red soil.

**Figure 1:**
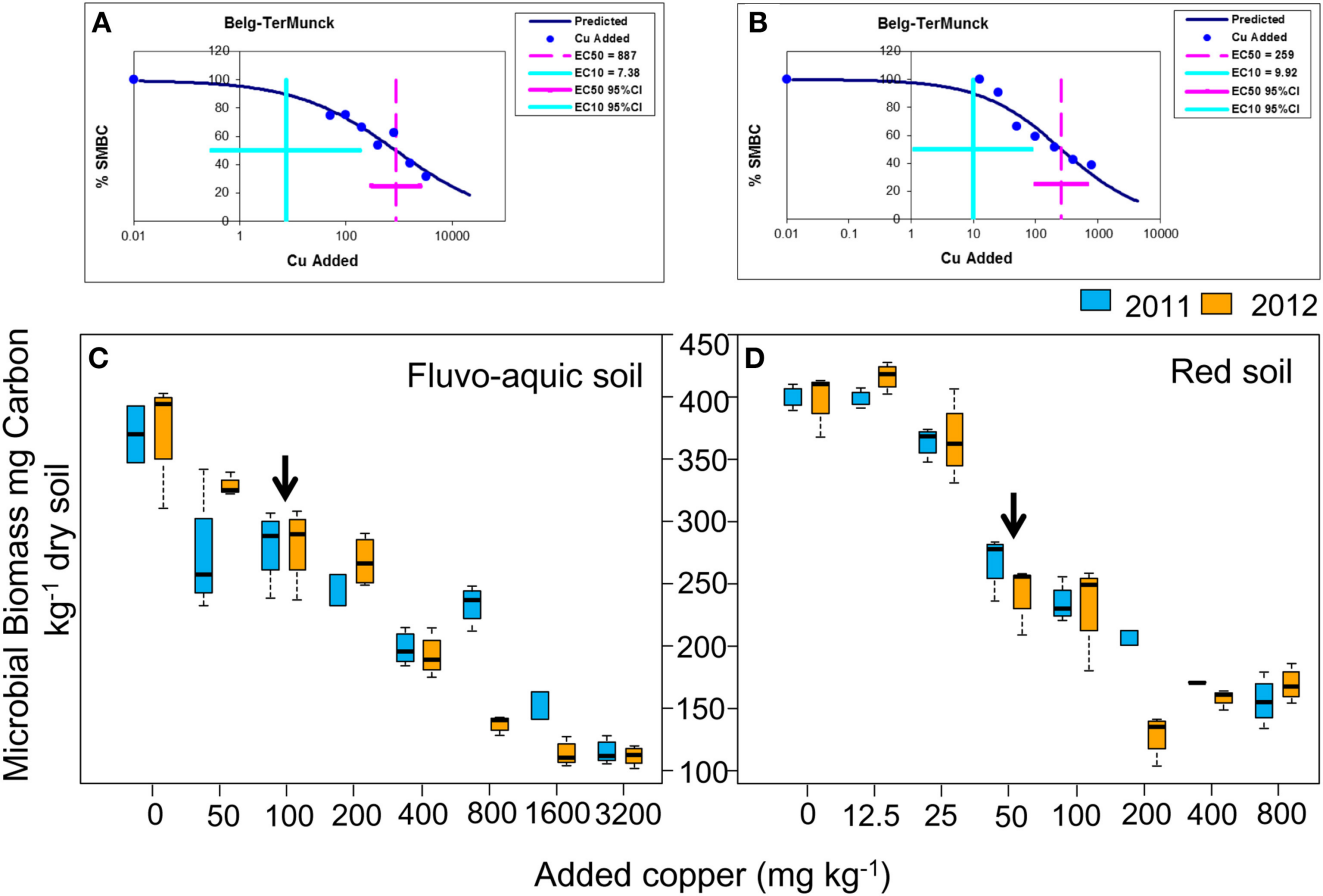
**Changes in SMBC across the eight copper concentrations in both years of the fluvo-aquic soil (C) and red soil (D), respectively.** The dose-response curves between the SMBC and added copper concentrations in the fluvo-aquic soil **(A)** and red soil **(B)**, respectively. The vertical pink lines indicate the EC50 values and the horizontal ones indicate 95% of the confidence intervals in **(A,B)**. The vertical blue lines indicate the EC10 values and the horizontal ones indicate 95% of the confidence intervals in **(A,B)**. The black arrows in **(C,D)** indicate the significant decreasing points of the SMBC.

SMBC was highly affected by copper contamination (Figure 1). In the fluvo-aquic soil, SMBC significantly decreased from 370.2 ± 16.1 mg C kg^−1^ soil in the control treatments to 111.4 ± 5.6 in the Cu3200 treatments (Figure 1C). The dose-effect relationships between the SMBC and the added copper concentrations were perfectly fitted by the logistic equation in both years (*P* < 0.01), and the toxicity threshold (EC50 values) was 887 ± 111 added Cu mg kg^−1^ soil (Figure 1A). Similar decreasing patterns were also observed in the red soil, with SMBC significantly reduced from 400.2 ± 6.4 mg C kg^−1^ soil in the Cu0 treatments to 170.1 ± 10.1 in the Cu800 treatments (Figure 1D). Further lower SMBC value was occasionally recorded in the Cu200 treatment. The significant dose-effect relationships between the SMBC and the added copper concentrations could also be observed in 2 years with the toxicity threshold (EC50 values) of 259 ± 37 added Cu mg kg^−1^ soil (Figure 1B) (*P* < 0.01). Spearman's rank analysis found significantly negative relationships between SMBC and copper concentrations in both soils.

### Differentiations in the bacterial abundance and diversity

**Figure 2:**
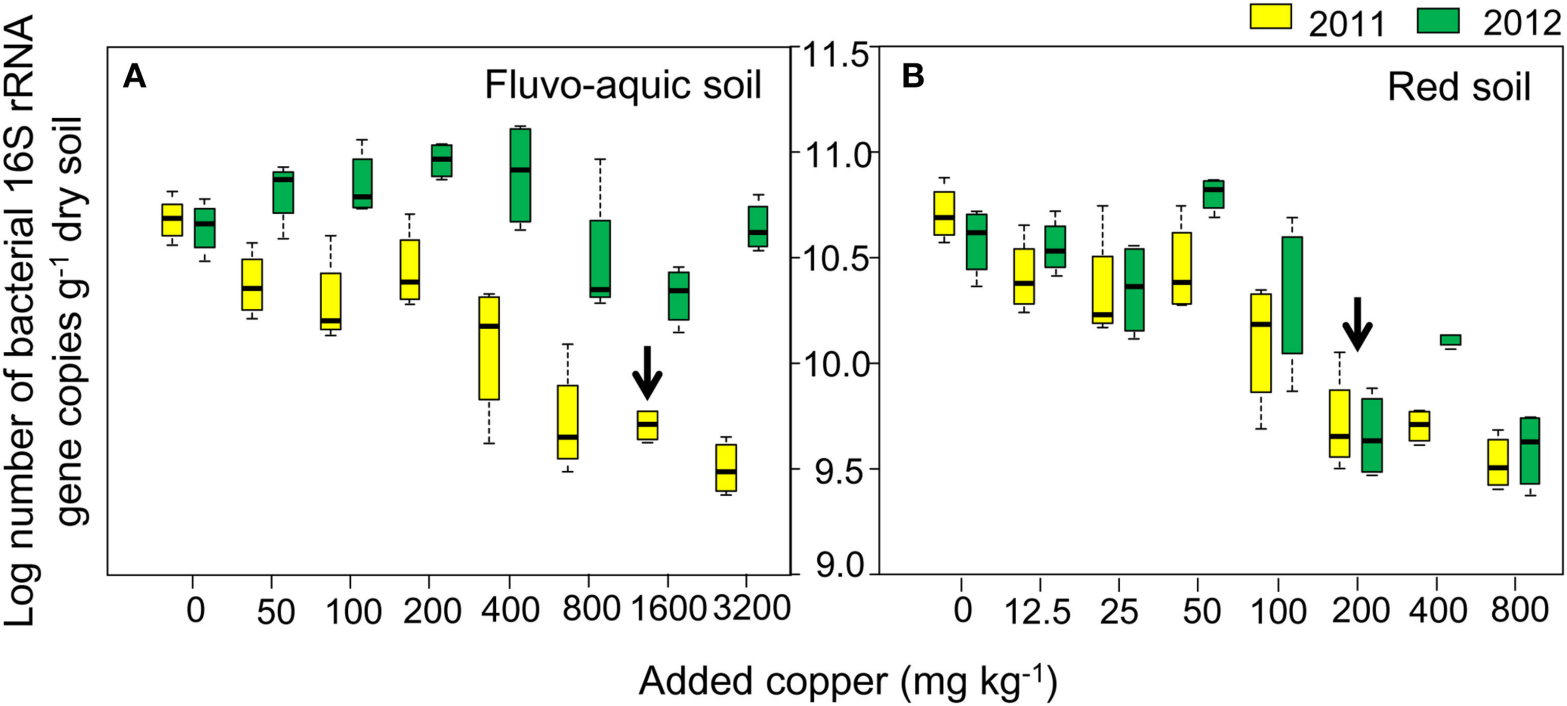
**Log-transformed abundance of the bacterial 16S rRNA gene across the eight copper concentrations in both years of the fluvo-aquic soil (A) and red soil (B), respectively.** The black arrows in **(A,B)** indicate the significant decreasing points of the abundance.

In the fluvo-aquic soil, copper additions significantly decreased the bacterial 16S rRNA gene abundance from 4.93 ± 0.64 × 10^10^ copies g^−1^ soil in Cu0 to 3.36 ± 0.58 × 10^9^ copies g^−1^ soil in Cu3200 in 2011, but its change in 2012 was much marginal, with a significantly lower value of 2.19 × 10^10^ copies g^−1^ soil recorded in Cu1600 (Figure 2A). In the red soil, the bacterial abundance gradually declined from 3.99 ± 0.72 × 10^10^ copies g^−1^ soil in Cu0 to 4.11 ± 0.82 × 10^9^ in Cu800 in both years (Figure 2B). Spearman's rank analysis revealed a significantly negative correlation between the bacterial abundance with copper concentrations in both soils.

**Figure 3:**
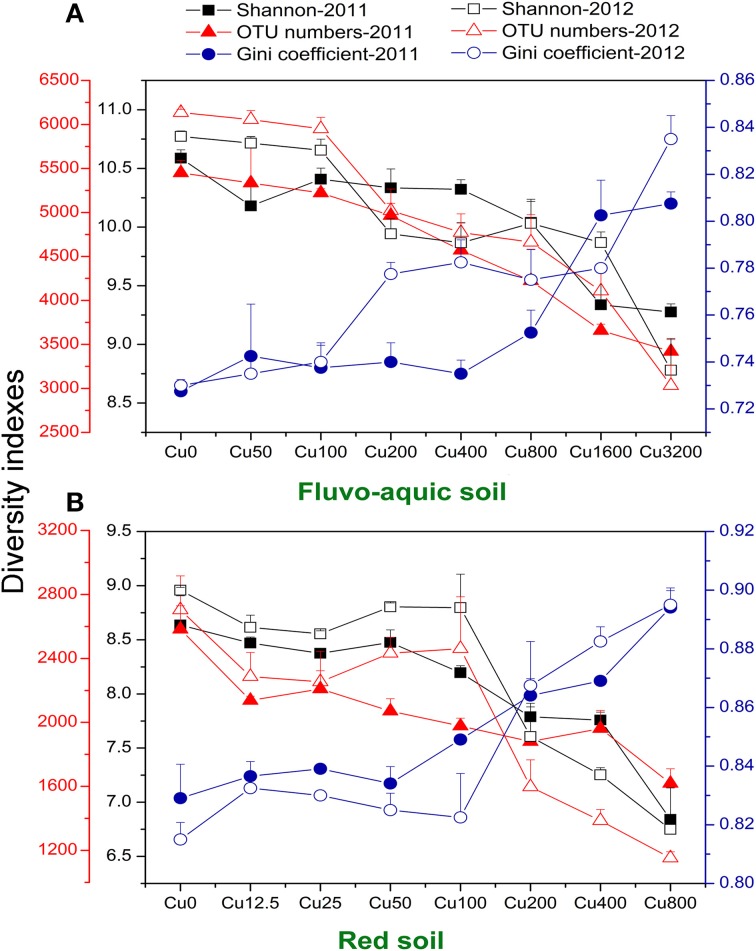
**Variations of soil microbial diversity along the copper gradients in both years of the fluvo-aquic soil (A), and red soil (B).** The dark blue y-axis indicates the Gini coefficient. The red and black y-axes indicates the OTU numbers and Shannon index, respectively. Error bars represent standard errors (*n* = 4).

The bacterial diversity was characterized by calculating evenness (Gini coefficient) and richness (OTU numbers and Shannon index at a 97% identity threshold). Gini coefficients gradually increased along the increasing copper concentrations, with the highest values found in treatments having the highest copper amendments in both years of the both soils (Figures 3A,B). OTU numbers and Shannon index showed similar declining trends in both soils, with continuously reduced values observed along the increasing copper levels (Figures 3A,B). The sharply increasing or decreasing points were 800 and 200 added Cu mg kg^−1^ in the fluvo-aquic soil and red soil, respectively. Significantly positive correlations between Gini coefficients and copper concentrations were found in both soils, while OTU numbers and Shannon index were significantly and negatively correlated with copper concentrations. OTU numbers and Shannon index in the fluvo-aquic soil were significantly higher than that in the red soil while the Gini coefficient displayed a reverse tendency, that is, the diversity was higher in the fluvo-aquic soil. Soil properties such as pH, H_2_O, SOM significantly determined the differences and pH explained 67% of the variance (*P* < 0.01).

### Community profiling of the bacterial community by illumina sequencing

**Figure 4:**
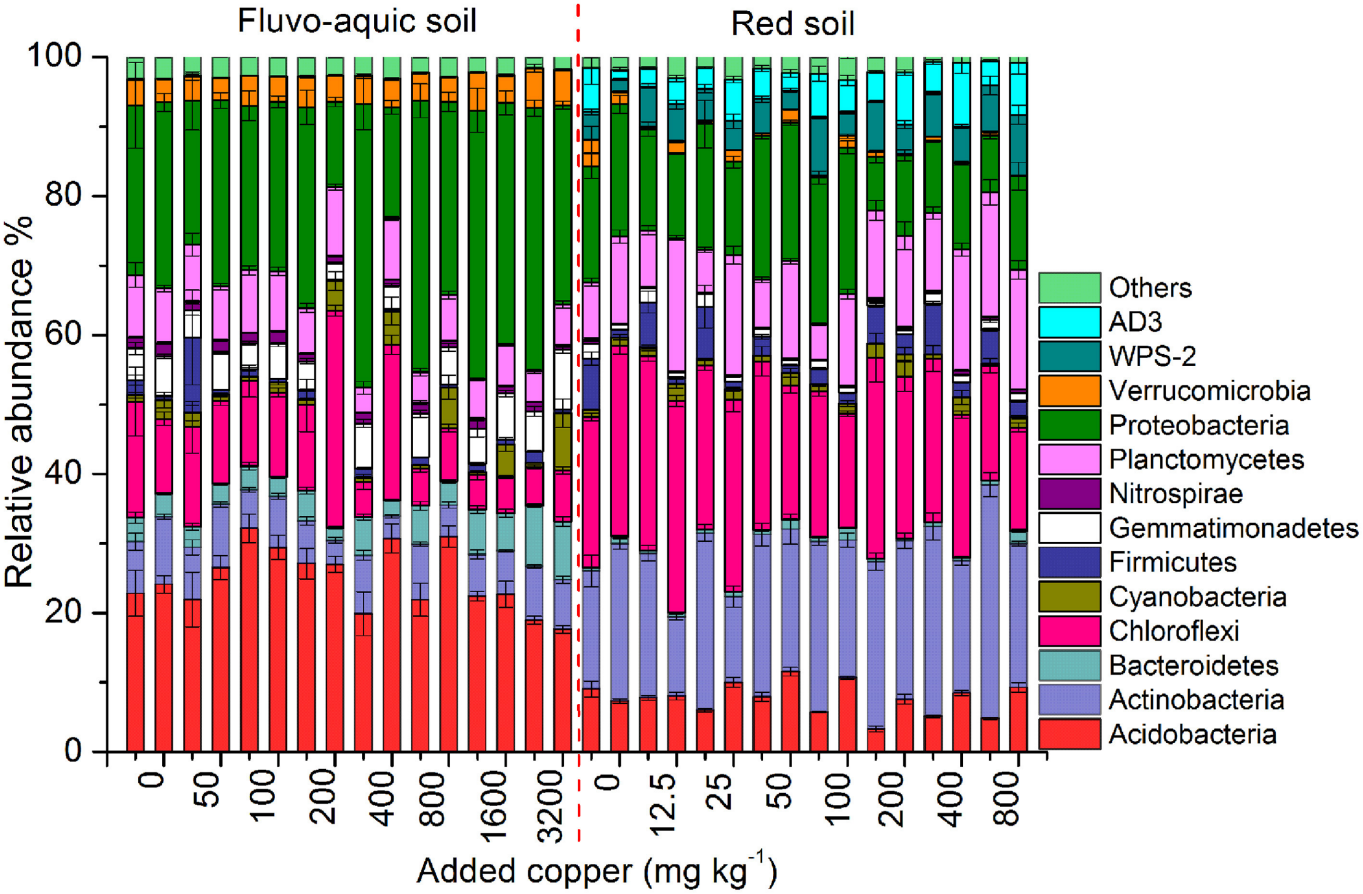
**Changes in the bacterial community compositions across the different copper concentrations in both years of the fluvo-aquic soil and red soil.** Error bars represent standard errors (*n* = 4). Here the two bars for each concentration represent the relative abundance of different groups in 2011 and 2012, respectively.

Across all the 128 samples examined, the high-throughput sequencing yielded 15,472,163 high-quality bacterial 16S rRNA gene sequences, and the minimum sequence number for individual sample was 47,721. The bacterial community in the fluvo-aquic soil was highly divergent from that in the red soil (Figure 4). *Proteobacteria* (12.24–40.84%), *Acidobacteria* (17.66–32.17%), *Chloroflexi* (4.96–31.29%), *Actinobacteria* (3.22–9.73%), *Planctomycetes*(3.60–9.93%), *Bacteroidetes* (1.79–8.76%), *Gemmatimonadetes* (2.43–8.52%) and *Verrucomicrobia* (3.25–5.74%) were the dominant phyla in the fluvo-aquic soil. However, the bacterial assemblages in the red soil were dominated by *Actinobacteria* (11.40–33.61%), *Chloroflexi* (14.88–30.36%), *Proteobacteria* (7.70–21.13%), *Planctomycetes* (5.09–19.18%), *Acidobacteria* (3.33–11.57%), *AD3* (1.47–9.20%), *WPS-2* (1.71–8.75%), and *Firmicutes* (1.11–7.57%). Compared with the fluvo-aquic soil, the relative abundances of *AD3*, *WPS-2*, *Actinobacteria*, *Chloroflexi*, *Firmicutes*, and *Planctomycetes* were significantly higher in the red soil. In contrast, the proportions of *Acidobacteria*, *Bacteroidetes*, *Gemmatimonadetes*, *Proteobacteria*, and *Verrucomicrobia* were significantly lower in the red soil than those in the fluvo-aquic soil (Figure 4). Soil properties such as pH, H_2_O and SOM significantly affected bacterial community compositions, and pH explained 93% of the differences (*P* < 0.01).

**Figure 5:**
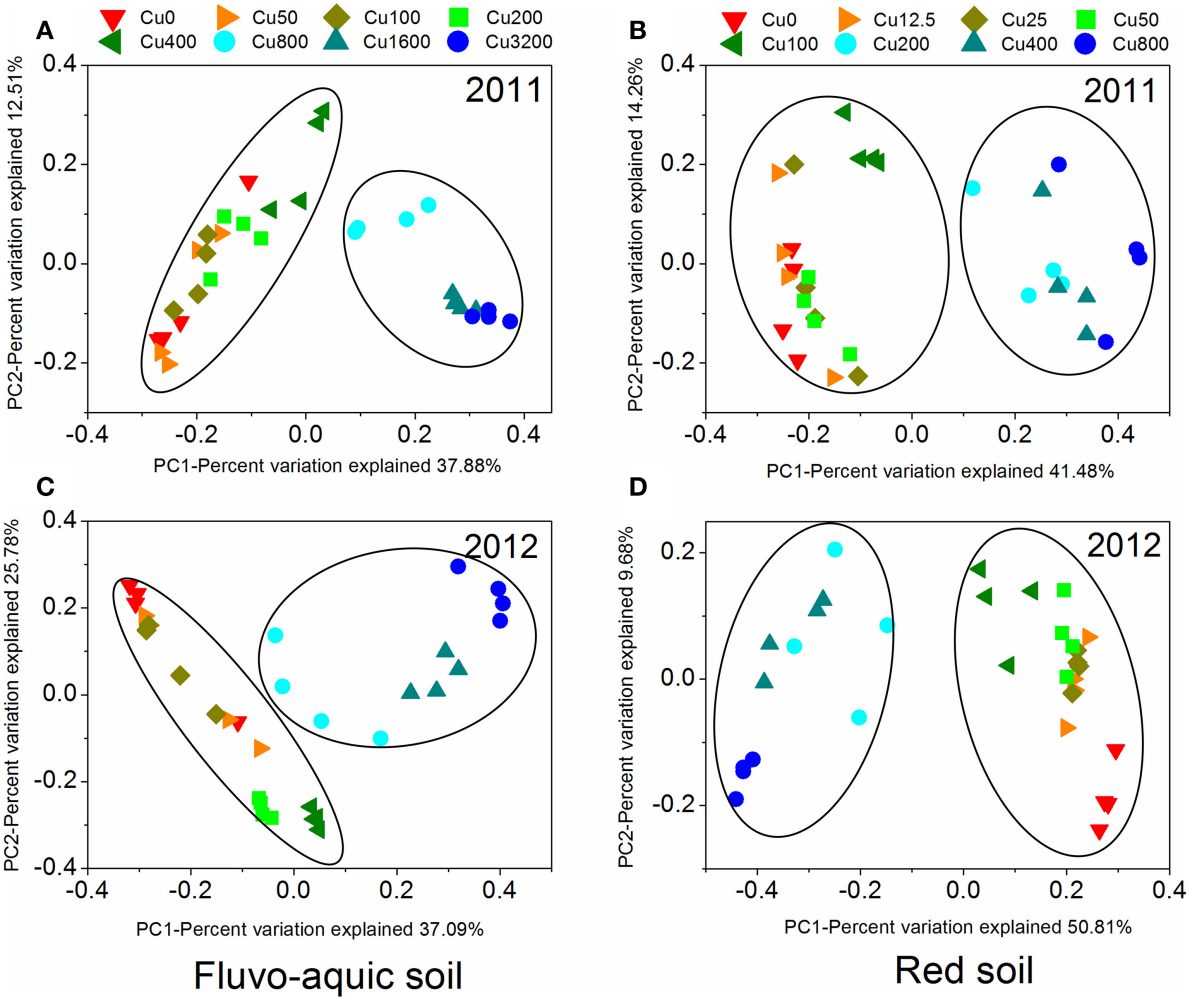
**The principal coordinate analysis (PCoA) derived from the Bray-Curtis dissimilarity matrices based on the 97% OTU level of the bacterial community compositions across different copper treatments in the fluvo-aquic soil (A and C) and red soil (B and D) at 2 sampling years**.

**Figure 6:**
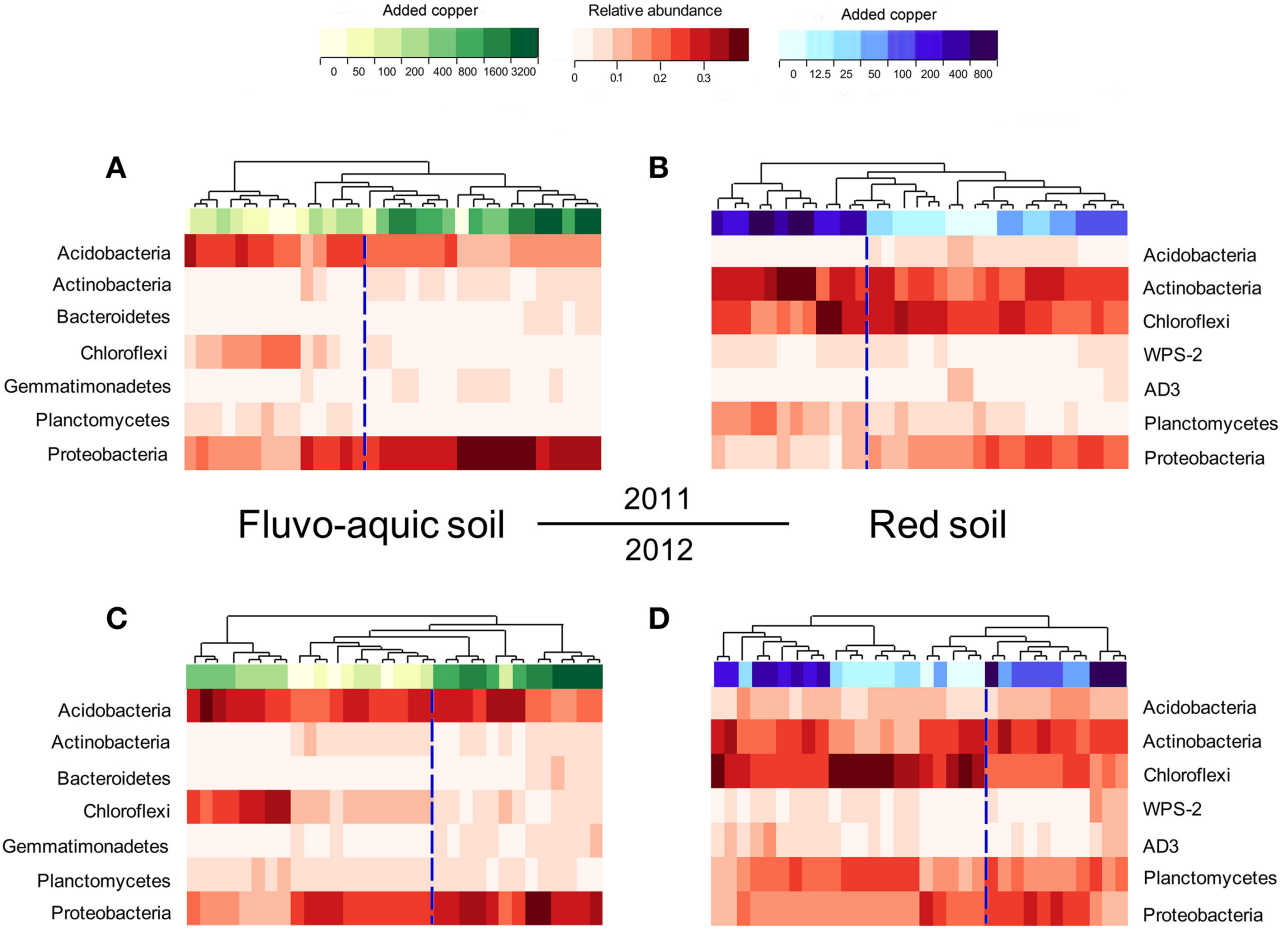
**Heat maps showing the shifts of the bacterial community compositions across the eight copper treatments in the fluvo-aquic soil in 2011 (A) and 2012 (C), and in the red soil in 2011 (B) and 2012 (D)**.

PerMANOVA analysis showed that the bacterial communities in treatments with ≥800 mg copper kg^−1^ soil could be obviously separated from those in treatments with lower copper concentrations in both years of the fluvo-aquic soil (Figures 5A,C). This finding was further corroborated by the partitioning effects of different copper concentration categories on the dominant bacterial phylum in the heat maps (Figures 6A,C). The differentiations of these major groups along the copper gradients were quite consistent between both years. The relative abundances of *Proteobacteria, Actinobacteria*, *Gemmatimonadetes*, and *Bacteroidetes* were observed to clearly increase with the increasing copper concentrations, whereas the shifts of *Acidobacteria*, *Chloroflexi*, and *Planctomycetes* were in reverse.

Similar to the fluvo-aquic soil, the bacterial communities in both years of the red soil were partitioned into two clusters: one from treatments with ≥200 mg copper kg^−1^ soil, and another from treatments with <200 mg copper kg^−1^ soil (PerMANOVA analysis, Figures 5B,D Supplementary Figure 1). The significant impacts of copper contamination on the bacterial community compositions were further supported by clustering of the dominant bacterial phylum corresponding to different copper levels in the heat maps (Figures 6B,D). Similar patterns in shifts of these major bacterial groups along the copper gradients were observed in 2011 and 2012. Generally, *Actinobacteria*, *WPS-2*, and *Planctomycetes* tended to have higher relative abundances at higher copper levels, *Proteobacteria* were more abundant at medium copper levels, while *Chloroflexi* showed higher frequencies at lower copper levels and reached the lowest at Cu800 in both years. *Acidobacteria* and *AD3* tended to be higher at lower copper levels in 2011, but had wider span across different copper levels in 2012.

### Relationships between the bacterial abundance, diversity, community composition with SMBC

Spearman's rank analyses found a significantly positive correlation between the bacterial abundance and SMBC under the copper contamination in both soils. Intriguingly, Gini coefficients had significantly negative correlations with SMBC, while OTU numbers and Shannon index had positive relationships with SMBC in both copper-contaminated soils. Moreover, the relative abundance of *Chloroflexi* was significantly and positively correlated with SMBC in both copper-contaminated soils (Supplementary Table 1).

**Figure 7:**
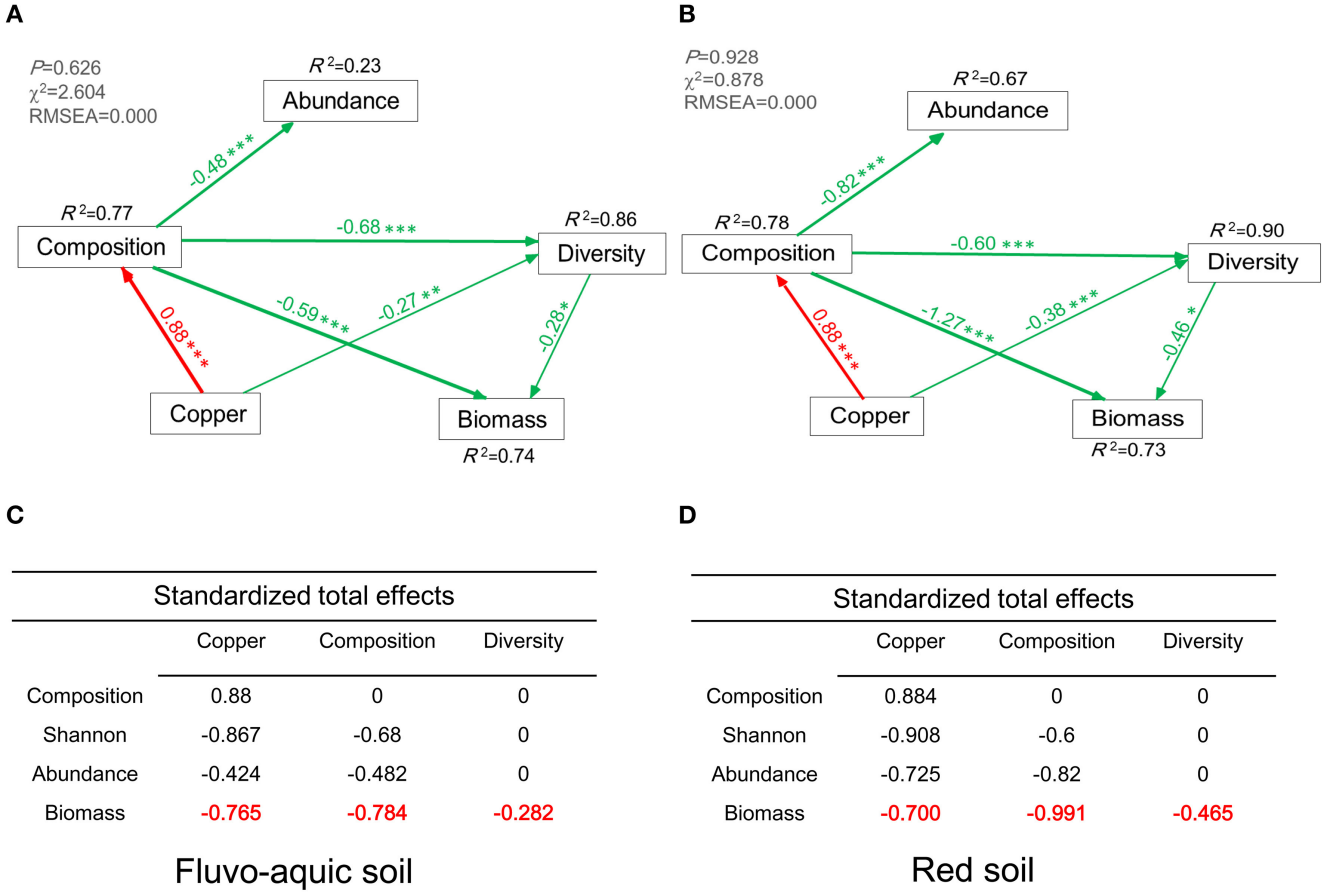
**Structural Equation Model (SEM) showing the causal relationships among bacterial abundance, diversity, biomass, community composition, and copper concentrations in the fluvo-aquic soil (A) and red soil (B).** The final model fits the data well **(A)**: maximum likelihood, χ^2^ = 2.604, *df* = 4, *P* = 0.626; goodness-of-fit index (GFI) = 0.984; Akaike Information Criteria (AIC) = 24.60; root square mean errors of approximation (RMSEA) = 0.000. **(B)**: maximum likelihood, χ^2^ = 0.878, *df* = 4, *P* = 0.928; goodness-of-fit index (GFI) = 0.994; Akaike Information Criteria (AIC) = 22.88; root square mean errors of approximation (RMSEA) = 0.000. Red and green lines indicate positive and negative pathways, respectively. Width of the line indicates the strength of the relationships. Standardized total effects (direct and indirect effects) were derived from the structural equation modeling **(C,D)**. ^*^P < 0.05, ^**^P < 0.01, ^***^P < 0.001.

The final SEM explained 74 and 73% of the variation in microbial biomass in the two soils, respectively (Figures 7A,B). Bacterial community composition and diversity showed directly negative effects on the microbial biomass in the fluvo-aquic soil (λ = −0.59, *P* < 0.001; λ = −0.28, *P* < 0.05, Figure 7A), and the similar negative effects were observed in the red soil (λ = −1.27, *P* < 0.001; λ = −0.46, *P* < 0.05, Figure 7B). Compared with these direct effects, copper concentrations indirectly affected the microbial biomass by influencing bacterial community composition and diversity (λ = −0.765, Figure 7C; λ = −0.700, Figure 7D). The total effects of bacterial community composition and diversity on the microbial biomass were also displayed in Figures 7C,D.

## Discussion

### Similar responses of the bacterial communities in two contrasting soils to the copper contamination

Although the long-term effects of copper contamination on microbial communities have been investigated in some studies (Macdonald et al., 2008; Singh et al., 2014), the responses of microbial community composition and function to copper contaminations differed across various sites and no firm conclusions had been drawn. Soil type is regarded as a key determinant of microbial communities (Drenovsky et al., 2010), and differences in soil basic characteristics can induce different microbial assemblages. As evidenced in this study, significant differences in bacterial communities including diversity and compositions between the two soils might be ascribed to the differences in soil properties especially pH. Our results showed that the relative abundances of *Gemmatimonadetes*, *Proteobacteria*, *Bacteroidetes*, and *Verrucomicrobia* were higher in the neutral/alkaline fluvo-aquic soil, which were in agreement with previous studies that these groups were favored under high pH (7~8) conditions (He et al., 2012; Fierer et al., 2013; Marcina et al., 2013). Interestingly, we also found that the relative abundance of *Acidobacteria* was higher in the fluvo-aquic soil than that in the red soil. According to the study of Jones et al. (2009), the distribution pattern of *Acidobacteria* was mainly regulated by soil pH, and different subgroups might respond differently to soil pH. In our study, the major subgroup of *Acidobacteria* in the fluvo-aquic soil belonged to subgroup 6, and this subgroup was reported to be prevailing in the high pH and low SOM environment (Zimmermann et al., 2005; Jones et al., 2009) which was similar to the characteristics of the examined fluvo-aquic soil. The diversity of soil bacteria is also dependent on soil pH (Hu et al., 2013; Marcina et al., 2013). In our study the bacterial diversity in the red soil was lower than that in the fluvo-aquic soil, which was corroborated by the finding that the bacterial diversity of acidic soil (pH 4~5) was much lower compared with the neutral soils (pH 7~8) (Fierer and Jackson, 2006). Based on the data from these long-term experimental sites, the soil pH values varied slightly between 2007 and 2011/2012, indicating that added copper did not significantly affect the soil pH.

The obvious differences of the thresholds between two soils were also found in our study, indicating the different EC50 values characterized by the SMBC and the thresholds that bacterial community compositions significantly separated across different copper concentrations in two soils. The toxicity thresholds characterized by SMBC obtained from the fluvo-aquic soil were significantly higher than those from the red soil, in addition, significant changes of the bacterial community compositions were induced by 800 added Cu mg kg^−1^ soil in the fluvo-aquic soil, and 200 mg kg^−1^ soil in the red soil (Figure 5). The probable reason could be ascribed to the higher pH in the alkaline fluvo-aquic soil, indicating that much of the Cu^2+^ could be precipitated as Cu_2_(OH)_2_CO_3_ (malachite) and Cu(OH)_2_ (Ma et al., 2006).

Although the two agricultural soils encompassed contrasting bacterial communities, the bacterial community compositions displayed consistent changes in response to the increasing copper gradients in both soils (Figure 5). Macdonald et al. (2011) found a similar response of bacterial, archaeal, and fungal community structures to copper and zinc contaminations in arable and grassland soils. However, apart from the community compositions, whether the soil microbial abundance, diversity and function all together respond similarly to the copper contamination across various sites remains unknown. Although the site effect was assumed to be the strongest determinant (Macdonald et al., 2011), our results did show a consistent shift in microbial community structure, and a consistent reduction of bacterial abundance, diversity and function, represented by SMBC, along the increasing copper gradients in both years of both soils. Therefore, our study provides strong evidence that the effects of copper contamination on bacterial communities are consistent across time and space (Singh et al., 2014).

### Critical copper-sensitive and -tolerant bacterial groups identified in the long-term field studies

One objective of our work was to identify key bacterial groups that might be responsive to copper contamination. It is likely that different microbial groups inhabit different niches within an ecosystem, and therefore differ in their sensitivity to copper contamination (Giller et al., 1998). Progressive shifts of the bacterial community compositions induced by the copper gradients were observed in our study, implying the dose-related effects as previously suggested (Brandt et al., 2010; Macdonald et al., 2011). The shifts might be explained by that some tolerant phylotypes were selected while sensitive ones were obviously reduced (Singh et al., 2014), and the adaption mechanism of these groups was attributed to different activities for biosorption, bioprecipitation, extracellular sequestration and chelation (Haferburg and Kothe, 2007). Specifically, we found that *Actinobacteria* was a copper tolerant group whereas *Acidobacteria* and *Chloroflexi* were copper sensitive groups in both soils.

A higher copper sensitivity of *Acidobacteria* has been reported in previous studies (Wakelin et al., 2010; Macdonald et al., 2011). *Acidobacteria* could thrive in soils with totally different physical and chemical characteristics and contribute to the terrestrial carbon cycle especially carbon storage (Trivedi et al., 2013), hence their apparent sensitivity to copper may be helpful to evaluate the impacts of copper on below-ground microorganisms and the relevant functioning. *Chloroflexi*, as a dominant group in both soils in our study, involved in carbon (Hug et al., 2013) cycling in the subsurface and exhibited copper sensitivity, which might be recognized as a new copper indicator. The tolerance of *Actinobacteria* to copper contamination has been reported in numerous studies (Haferburg and Kothe, 2007; Sun et al., 2010), and the resistance mechanisms could be explained by the active metal efflux transport systems or by complexation with metal chelating substances for limiting free ions of metals within the cell (Haferburg and Kothe, 2007). As for a microbial community, species with high resistance to the heavy metal contamination might compensate the loss of other species affected by the contamination, which guaranteed the stable community (Awasthi et al., 2014).

### Significant influences of the copper contamination on soil bacterial communities and their potential effects on microbial functioning

The significant loss of microbial diversity induced by copper has been reported in the studies of contaminated soil (Singh et al., 2014). More and more studies reported that microbial assemblages respond sensitively to copper contamination via changes in community composition and/or function (Macdonald et al., 2011; Griffiths and Philippot, 2012; Li et al., 2014a). As evidenced in this study, despite the different soil types, we found that bacterial diversity significantly decreased along the copper gradients in both agricultural soils, and the apparent shifts of bacterial community compositions were also observed. The microbial biomass accumulation provides important information about community functioning and could be regarded as a functional indicator (Shade et al., 2012; Awasthi et al., 2014). In our study, the microbial biomass, as presented by SMBC, also decreased with the increasing copper concentrations. However, the bacterial abundance did not show a significantly decreasing tendency as the SMBC. The most probable reason might be explained by the variations of fungi communities which also contribute to the productivity of the SMBC.

The role of biodiversity acting as ecological insurance is crucial for maintaining the ecosystem functioning under environmental fluctuations (Loreau and de Mazancourt, 2013). As for the bacterial diversity in this study, the richness and evenness were positively correlated with SMBC in both copper contaminated soils, and this finding was in agreement with a strongly positive linear relationship between soil biodiversity and ecosystem multifunctionality (Wagg et al., 2014). The changes in soil microbial community compositions also influenced ecosystem processes related to nutrient cycling (Wagg et al., 2014), and our findings that the relative abundance of *Chloroflexi* was positively correlated with SMBC supported this hypothesis. *Chloroflexi* are also considered to be involved in carbon cycling such as CO_2_ fixation in the subsurface (Hug et al., 2013), which contribute to the production of microbial biomass carbon. Moreover, the thresholds that bacterial diversity and community composition obviously changed were 800 and 200 added mg kg^−1^ copper in the fluvo-aquic soil and red soil, respectively, which were similar to the toxicity thresholds (EC50 values) by fitting the dose-effect relationships between SMBC and added copper concentrations. SEM analysis further established the relationships between the bacterial communities and the microbial biomass, which demonstrated that the shifts in bacterial community compositions and diversity could have largely determined the variations of microbial biomass in both soils. This finding further supported that the changes in microbial communities could indeed influence the microbes-mediated function.

Overall, these results provide remarkable evidence that both the shifts of diversity and composition of bacterial assembly might result in the changes of SMBC under the copper contamination, and they agree with that highly diverse communities are prone to maintaining higher community productivity (Awasthi et al., 2014). Biodiversity loss negatively affecting the ecosystem services has already been recognized in the above-ground communities (Zavaleta et al., 2010), recent research gradually extends to the below-ground environment, suggesting that loss of soil biodiversity and changes in soil community composition impact the performance of overall ecosystem and threaten ecosystem multifunctionality and sustainability (Wagg et al., 2014). Our consistent findings in contrasting soil types support this conclusion, and this study might also contribute to the work focusing on the effects of diversity and compositions of soil microbial communities on ecosystem functions enduring the heavy metal contamination. However, there was an obvious limitation in our study that we only measured the microbial biomass and did not investigate multiple microbial functions, therefore, more comprehensive work is desirable in the future.

In conclusion, by tracking the detailed feedback responses of soil bacteria to copper contamination in two different soil types, we provide critical field-based evidence that copper contamination over the long-term could lead to remarkable changes in the bacterial abundance and compositions, and significant losses of diversity. These taxonomic changes might result in alternations of major functional capacities, as represented by the consistent reduction in the microbial biomass production under the copper contamination. The copper tolerant guilds identified in this study could serve as a basis for future discovery of microbial assemblages with bioremediation potential and strong adaptability to be utilized in copper contaminated sites. Our findings caution the negative impacts on terrestrial ecosystem security brought about by intensive human activities, and highlight the necessity of preserving belowground biodiversity for sustainable ecosystem functioning.

### Conflict of interest statement

The authors declare that the research was conducted in the absence of any commercial or financial relationships that could be construed as a potential conflict of interest.
