# Supplementary material for: Field-based evidence for consistent responses of bacterial communities to copper contamination in two contrasting agricultural soils
Source: Front Microbiol. 2015 Feb 2;6:31. doi: 10.3389/fmicb.2015.00031 (PMC4313605; doi:10.3389/fmicb.2015.00031)

Supplementary Material

Field-based evidence for consistent responses of bacterial communities to copper contaminations in two contrasting agricultural soils

Jing Li [1,2], Yi-Bing Ma[3], Hang-Wei Hu [4], Jun-Tao Wang [1,2] , Yu-Rong Liu [1], Ji-Zheng He [1,4]*

[1]State Key Laboratory of Urban and Regional Ecology, Research Center for Eco-Environmental Sciences, Chinese Academy of Sciences, Beijing, China

[2]University of Chinese Academy of Sciences, Beijing, China

[3]National Soil Fertility and Fertilizer Effects Long-term Monitoring Network, Institute of Agricultural Resources and Regional Planning, Chinese Academy of Agricultural Sciences, Beijing, China

[4]Faculty of Veterinary and Agricultural Sciences, The University of Melbourne, Parkville VIC 3010, Australia

Correspondence:
Dr. Ji-Zheng He
State Key Laboratory of Urban and Regional Ecology
Research Center for Eco-Environmental Sciences, Chinese Academy of Sciences
18 Shuangqing Road
Haidian District, Beijing 100085, China
jzhe@rcees.ac.cn or jizheng.he@unimelb.edu.au

Supplementary Figures and Tables

Supplementary Table 1 Spearman correlations between bacterial abundance, diversity, community composition and SMBC.

	Abundance	Diversity			Composition
		OTU numbers	Shannon index	Gini coefficient	Chloroflexi (%)
SMBC	r = 0.711<br>$P < 0.001$	r = 0.576<br>$P < 0.001$	r = 0.512<br>$P < 0.001$	r = -0.514<br>$P < 0.001$	r = 0.163<br>$P < 0.05$

Supplementary Figure 1 The Nonmetric Multidimensional Scaling (NMDS) derived from the Bray-Curtis dissimilarity matrices based on the 97% OTU level of the bacterial community compositions across different copper treatments in the red soil at two sampling years.

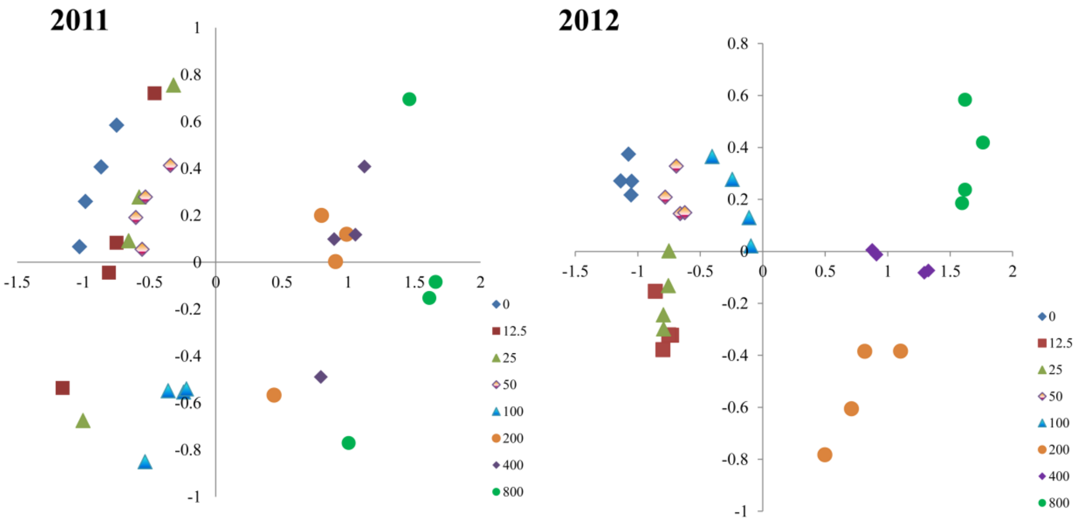

Supplement: Supplementary file 1 [file Presentation1.PDF]
